# Diversity of *Pneumocystis jirovecii* during Infection Revealed by Ultra-Deep Pyrosequencing

**DOI:** 10.3389/fmicb.2016.00733

**Published:** 2016-05-24

**Authors:** Alexandre Alanio, Maud Gits-Muselli, Séverine Mercier-Delarue, Françoise Dromer, Stéphane Bretagne

**Affiliations:** ^1^Laboratoire de Parasitologie-Mycologie, Groupe Hospitalier Saint-Louis-Lariboisière-Fernand-Widal, Assistance Publique Hôpitaux de Paris, Hôpital Saint-LouisParis, France; ^2^Université Paris Diderot, Sorbonne Paris CitéParis, France; ^3^Unité de Mycologie Moléculaire, Département de Mycologie, Centre National de Référence Mycoses Invasives et Antifongiques, Institut PasteurParis, France; ^4^Centre National de la Recherche Scientifique CNRS URA3012Paris, France; ^5^Laboratoire de Microbiologie, Groupe Hospitalier Saint-Louis-Lariboisière-Fernand-Widal, Assistance Publique Hôpitaux de Paris, Hôpital Saint-LouisParis, France

**Keywords:** ultra-deep pyrosequencing, *Pneumocystis jirovecii*, mixed infection, mitochondria, heteroplasmy, recombination

## Abstract

*Pneumocystis jirovecii* is an uncultivable fungal pathogen responsible for *Pneumocystis* pneumonia (PCP) in immunocompromised patients, the physiopathology of which is only partially understood. The diversity of the *Pneumocystis* strains associated with acute infection has mainly been studied by Sanger sequencing techniques precluding any identification of rare genetic events (< 20% frequency). We used next-generation sequencing to detect minority variants causing infection, and analyzed the complexity of the genomes of infection-causing *P. jirovecii*. Ultra-deep pyrosequencing (UDPS) of PCR amplicons of two nuclear target region [internal transcribed spacer 2 (ITS2) and dihydrofolate reductase (DHFR)] and one mitochondrial DNA target region [the mitochondrial ribosomal RNA large subunit gene (mtLSU)] was performed on 31 samples from 25 patients. UDPS revealed that almost all patients (*n* = 23/25, 92%) were infected with mixtures of strains. An analysis of repeated samples from six patients showed that the proportion of each variant change significantly (by up to 30%) over time on treatment in three of these patients. A comparison of mitochondrial and nuclear UDPS data revealed heteroplasmy in *P. jirovecii*. The recognition site for the homing endonuclease I-SceI was recovered from the mtLSU gene, whereas its two conserved motifs of the enzyme were not. This suggests that heteroplasmy may result from recombination induced by unidentified homing endonucleases. This study sheds new light on the biology of *P. jirovecii* during infection. PCP results from infection not with a single microorganism, but with a complex mixture of different genotypes, the proportions of which change over time due to intricate selection and reinfection mechanisms that may differ between patients, treatments, and predisposing diseases.

## Introduction

*Pneumocystis jirovecii* is an ascomycete fungus that specifically infects humans (Cushion, 2010; Gigliotti and Wright, 2012) and causes *Pneumocystis* pneumonia (PCP), mostly in immunocompromised patients, including HIV-positive, solid organ transplant, and cancer/hematology patients, but also in adults and children with various other underlying conditions (Pagano et al., 2002; Roblot et al., 2003; Catherinot et al., 2010; Wissmann et al., 2010; Reid et al., 2011; Mori and Sugimoto, 2012; Tasaka and Tokuda, 2012). *P. jirovecii* thrives at the surface of alveolar pneumocytes, but is unable to grow on standard artificial media. It can, however, be amplified *in vitro* in an air-liquid interface culture system (Schildgen et al., 2014). These characteristics have made it difficult to study its genetic diversity, complexity, and evolution in humans. In addition, the genome sequence of *P. jirovecii* is only recently publicly available (Cissé et al., 2012; Cushion and Keely, 2013).

Since the 1990s, specific tools have been used to study the genetic diversity of *P. jirovecii*. In addition to cloning and sequencing (Tsolaki et al., 1996), single-strand conformation polymorphism (Hauser et al., 2001; Hauser, 2004), single-nucleotide extension assays (Esteves et al., 2011; Alanio et al., 2015), multi-locus sequencing typing (MLST) (Maitte et al., 2013), and short tandem repeat (STR)-based genotyping methods (Parobek et al., 2014; Gits-Muselli et al., 2015) have shown that mixtures of genotypes, differing in at nuclear or mitochondrial loci, are present during active infection. It has been estimated that up to 70% of patients with PCP harbor multiple genotypes, as assessed in various populations and by diverse techniques targeting nuclear or mitochondrial genes (Hauser et al., 2001; Parobek et al., 2014; Gits-Muselli et al., 2015). Studies based on Sanger sequencing have also reported the presence of mixed *P. jirovecii* sequences, but in a lower proportion of patients (about 30%) due to the well-known poor sensitivity of the Sanger method for detecting minority alleles (Angulo et al., 2010).

The genetic diversity of *P. jirovecii* has mostly been investigated in epidemiological studies, in which little attention was paid to the differences between nuclear and mitochondrial markers. The mitochondrial ribosomal RNA large subunit gene is currently used as a PCR target for PCP diagnosis (Meliani et al., 2003; Alanio et al., 2011, 2016; Botterel et al., 2012) and for typing based on MLST (Maitte et al., 2013). However, the nuclear and mitochondrial genomes can behave differently. Indeed, mitochondrial DNA can undergo more recombination events and acquire more mutations over time than nuclear DNA (Fritsch et al., 2014). The mitochondrial genome of *P. jirovecii* has recently been sequenced and compared to those of other *Pneumocystis* species (Ma et al., 2013). Although the mitochondrial genome of *Pneumocystis carinii* and *Pneumocystis murina* are thought to be linear, that of *P. jirovecii* appears to consist of a double-stranded circular mitochondrial DNA (mtDNA) molecule (Ma et al., 2013) that is autonomously replicated, transcribed, and mostly encodes proteins involved in mitochondrial respiration (Chen and Butow, 2005). In fungi, the mitochondrial genome mostly displays uniparental inheritance (Basse, 2010) and is autonomously replicated and transcribed (Chatre and Ricchetti, 2014).

We therefore investigated the diversity of *P. jirovecii* sequences, using ultra-deep pyrosequencing (UDPS) to detect minority variants and to compare the dynamics of the mitochondrial and nuclear genomes in the context of infection.

## Materials and methods

### Ethics statement

Saint-Louis Hospital, Paris, France, is a 650-bed tertiary university hospital with major clinical activities in hematology, renal transplantation, and oncology. This study was a non-interventional study with no change in the usual procedures. Biological material and clinical data were obtained only for standard diagnostic following physicians' prescriptions with no specific sampling. According to the French Health Public Law (CSP Art L1121-1.1), such protocol does not require approval of an ethics committee and is exempted from specific informed consent application.

### Patients and samples

We studied a collection of 31 respiratory samples from 25 patients with PCP treated at our university hospital (Saint Louis Hospital, Paris, France). Patients and samples were selected randomly from a collection of sputum or BAL fluids from patients with PCP and a high load of fungal DNA (Cq < 30, Alanio et al., 2011). Diagnosis was based on clinical (pulmonary infection in immunocompromised patients), radiological (interstitial pneumonia, ground glass opacities), and biological findings (ascus observed on immunofluorescence analyses of smears of respiratory material). DNA was extracted as previously described (Alanio et al., 2011). Our real-time quantitative PCR assay confirmed the high fungal load in all samples (>1900 EqTr/ml) (Alanio et al., 2011). The median age of the patients was 50 years (range: 1–80 years), and the sex ratio (M/F) was 5.25. The patients were HIV-positive (*n* = 12, 48%), had hematological malignancies (*n* = 6, 24%), were kidney transplant recipients (*n* = 4, 16%) or had another cause of immunodeficiency (*n* = 3, 12%). Two samples were collected from each of six patients (12 samples) with a median interval between samples of 7.5 (1–18) days. All samples had already been genotype with a recently described short-tandem repeat (STR) genotyping assay (Gits-Muselli et al., 2015). The six STR markers used were evenly distributed in the nuclear genome, as shown by alignment with the *P. murina* genome sequence (Gits-Muselli et al., 2015). The limit of detection for mixed STR markers was shown experimentally to be 2% (Gits-Muselli et al., 2015).

### Selection of target genes and amplicons

We were able to analyze amplicons of up to 320 bp in length in specific assays. The 5.8S-ITS2-28S (*n* = 120), DHFR (*n* = 60), and mtLSU (*n* = 52) sequence hits collected from Nucleotide Blast (https://blast.ncbi.nlm.nih.gov/Blast.cgi?PAGE_TYPE=BlastSearch) were aligned, using Geneious 7.1 (Biomatters Ltd., New Zealand). We selected primers (Table S2) binding to conserved regions and allowing the amplification of sequences of about 300 bp in length, to maximize the number of polymorphic positions detected (Figures S1A–S3A). The primers were designed with Primer3web v 4.0.0 (http://primer3.ut.ee).

### Specific gene amplification by UDPS

Each PCR primer consisted of a specific adaptor (5′-CGTATCGCCTCCCTCGCGCCATCAG-3′ for forward primers and 5′CTATGCGCCTTGCCAGCCCGCTCAG-3′ for reverse primers), a sample-specific multiplex identifier (MID) sequence, and a specific amplification sequence for PCR (Table S2).

We obtained three amplicons for each sample. Each amplicon (22.5 μl) was diluted with an equal volume of water and purified with AMPure beads (72 μl), before quantification with a Qubit^®;^ ds DNA HS Assay kit (Life Technologies) on a Qubit 3.0 instrument. For each gene, the PCR amplicons from 16 samples were pooled in equimolar concentrations and used to prepare an equimolar mixture of 10^7^ molecules. This pool was subjected to clonal amplification on capture beads by emulsion PCR. In total, 500,000 beads enriched in DNA were deposited into the wells of a PicoTiter plate device and subjected to pyrosequencing in both directions with the GS Junior system (Roche). After a 10-h run, total reads were analyzed with GS amplicon variant analyzer (AVA) software (Roche), filtered and assigned, by demultiplexing, to the correct patient sample on the basis of MID correspondence.

### Analysis of UDPS data

AVA software (Roche) also aligned the generated sequence reads with the chosen reference sequence (JQ365725 for 5.8S-ITS2-28S, AF090368 for DHFR, JX499143 for mtLSU) and identified the proportion of polymorphism for each variable position in the sequences amplified. Insertions and deletions were not considered. Only already known SNPs were taken into account and haplotypes representing >0.5% of the sequences were selected for further analysis, to prevent the introduction of false polymorphic regions due to PCR errors (Brodin et al., 2013). Each individual combination of SNPs was selected and called haplotypes.

Reproducibility was determined by testing the same sample in two runs. The proportion of each of the variants sequenced varied by no more than 2%.

In all analyses, we studied one sample per patient (*n* = 25): the first sample collected. Specific analyses of repeated samples were performed separately.

### Screening for the presence of a homing endonuclease

We screened for the presence of conserved I-SceI motifs (GIGLILGDA and LAYWFMDDG) or motifs derived from this enzyme from *Taphrina deformans* (IVGLMLGDGY and LAVWISDDG) and for the recognition site of this enzyme (AAGTTACGCTAGGGATAACGGGGTAATATAGC) in the mitochondrial genome of *P. jirovecii* (JX499143), with Geneious v8.0 (Biomatters Ltd.).

### Graphs and statistical analysis

Sequence analysis graphs were generated with Geneious v8.0 (Biomatters Ltd.). Hierarchical clustering was performed by Pearson's correlation analysis and average linkage clustering, with MeV v4.6.1 open-source genomic analysis software (The TM4 Development Group) obtained from www.tm4.org (Saeed et al., 2006). All graphs were plotted and statistical analyses (*t*- tests, One-way ANOVA, chi-square) were performed with Prism software v.6.0 (Graphpad).

## Results

### UDPS detected numerous variant sequences

We used UDPS to analyze the diversity of three genetic sequences in 31 respiratory samples from 25 patients. We investigated two nuclear DNA target regions [partial 5.8S, internal transcribed spacer 2 and partial 28S gene (ITS2), GenBank accession n°JQ365725] and the [partial dihydrofolate reductase gene (DHFR), GenBank accession n°AF090368] and one mitochondrial DNA target region [partial mitochondrial ribosomal RNA large subunit gene (mtLSU), GenBank accession n°JX499143] (Figures S1A–S3A). The ITS2 locus was selected because this target DNA has already been used for genotyping *P. jirovecii* samples (de Boer et al., 2007; Le Gal et al., 2012) and is known to be present as a single copy in the *P. jirovecii* genome (Nahimana et al., 2000; Cissé et al., 2012; Cushion and Keely, 2013). The dihydrofolate reductase gene (DHFR, target gene of trimethoprim (diaminopyrimidine), one of the components of cotrimoxazole) was also selected because it was a single-copy gene and because mutations of this gene have already been described and potentially associated with the failure of cotrimoxazole prophylaxis (Nahimana et al., 2004; Queener et al., 2013). The mtLSU locus is the DNA target most widely used for diagnosis, it is known to be single-copy in the mitochondrial genome (Ma et al., 2013), with the mitochondria and mitochondrial genome being replicated several times within each organism. Two runs per gene were performed by multiplexing. After filtering, sequences were obtained and aligned with the corresponding reference sequence (with a mean of 6552 ± 3525, 8107 ± 2625, 4022 ± 1892 reads for ITS2, DHFR, and mtLSU, respectively).

**Figure 1:**
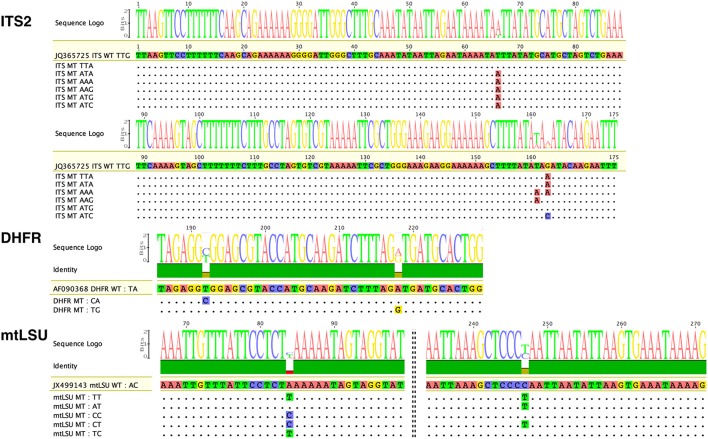
**Variants of the three ***Pneumocystis jirovecii*** target DNA regions observed in our collection of samples by ultra-deep pyrosequencing (UDPS)**. Analysis of all 31 samples led to the identification of seven variants for the internal transcribed spacer two region (ITS2), two for the partial dihydrofolate reductase gene (DHFR), and six for the mitochondrial ribosomal RNA large subunit gene (mtLSU), with respect to reference sequences (GenBank Accession number: JQ365725, AF090368, and JX499143, respectively). Of note, for ITS2, the numbering corresponds to the ITS2 region and not to that of the whole amplicon. For the ITS2 target, three polymorphic bases at positions 66, 161, and 163 of the ITS2 region resulted in the observation of seven variants: TTG, TTA, ATA, AAA, AAG, ATG, and ATC. For the DHFR gene, two polymorphic bases at positions 192 and 218 of the amplicon resulted in the observation of only three variants. For the mtLSU target, two polymorphic bases at positions 84 and 247 of the amplicon resulted in the observation of six variants: AG (WT), TT, AT, CC, CT, and TC. Dots indicate bases identical to the reference sequence. Polymorphic bases are shown in color. The size of the consensus base (sequence Logo) is proportional to the proportion of sequences polymorphic at the corresponding position. WT, wild-type sequence corresponding to the reference sequence; MT, mutated sequence corresponding to polymorphic variants.

Polymorphisms were observed at positions 135 (T135A), 230 (T230A), and 232 (G232A or G232C) of the 277 bp ITS2 amplicon, at positions 192 (T192C) and 218 (A218G) of the 300 bp DHFR amplicons, and at positions 84 (A84T or A84C) and 247 (C247T) of the 314-bp mtLSU amplicons (Figure 1, Table S1, Figures S1B–S3B). We used these polymorphisms to determine the haplotypes [combination of single nucleotide polymorphisms (SNPs)] of the 31 samples. Overall, seven variants (type of haplotypes) were found for ITS2 (TTG [WT], ATG, AAG, TAT, ATC, TTA, AAA), three for DHFR (TA [WT], CA, and TG) and six for mtLSU (AC [WT], TT, AT, CC, CT, TC) (Figure 1, Figures S1B–S3B).

**Figure 2:**
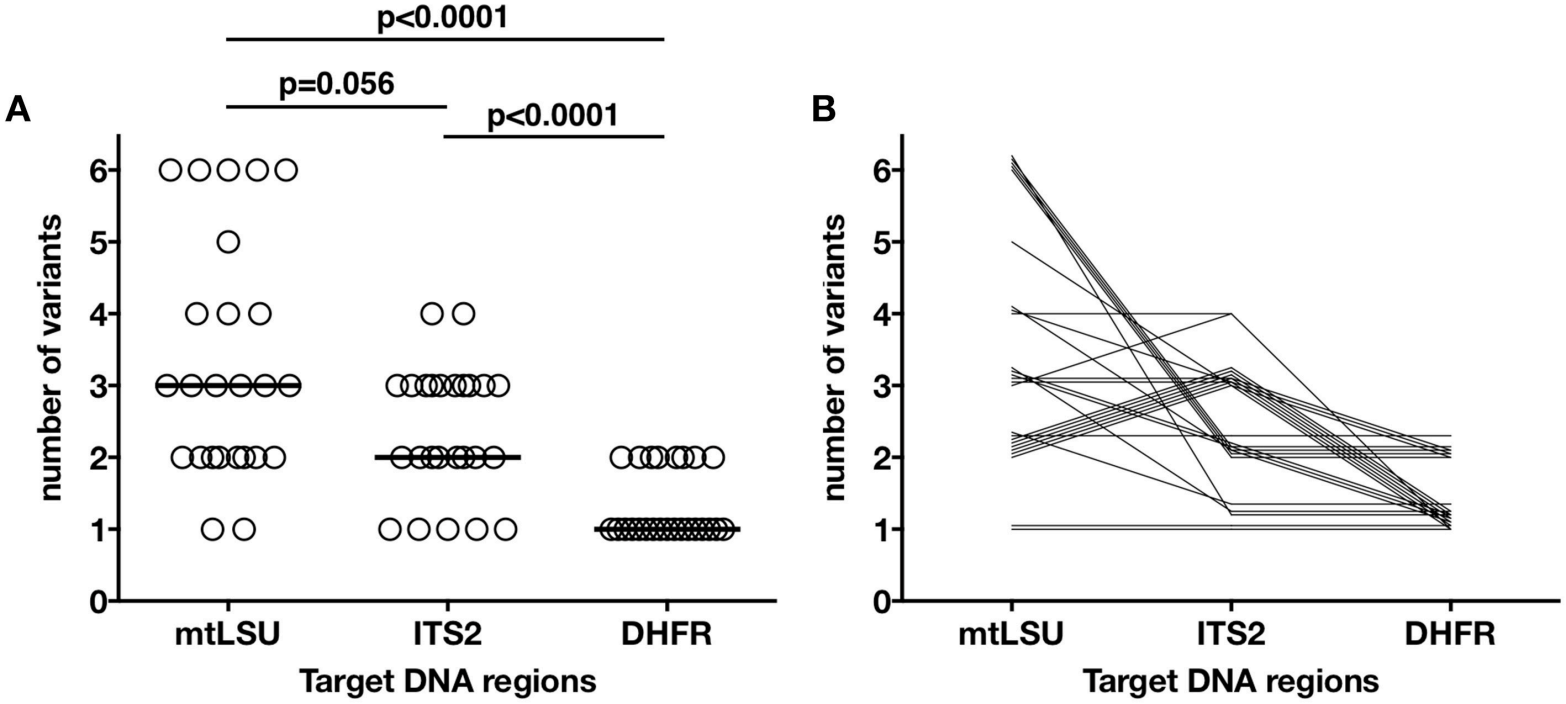
**Distribution of the number of variants found in each sample for the mtLSU, ITS2, and DHFR DNA targets**. Each of the 25 samples is represented as an independent dot **(A)**. The number of variants was significantly different between the three genes (one-way ANOVA, *p* < 0.0001), with mtLSU and ITS having significantly more variants than DHFR. The bar represents the median for each target region. Each of the samples is represented by a line linking the number of variants found for each of the three genes **(B)**. Similar patterns in the distribution of the variants can be seen for our 25 samples.

**Figure 3:**
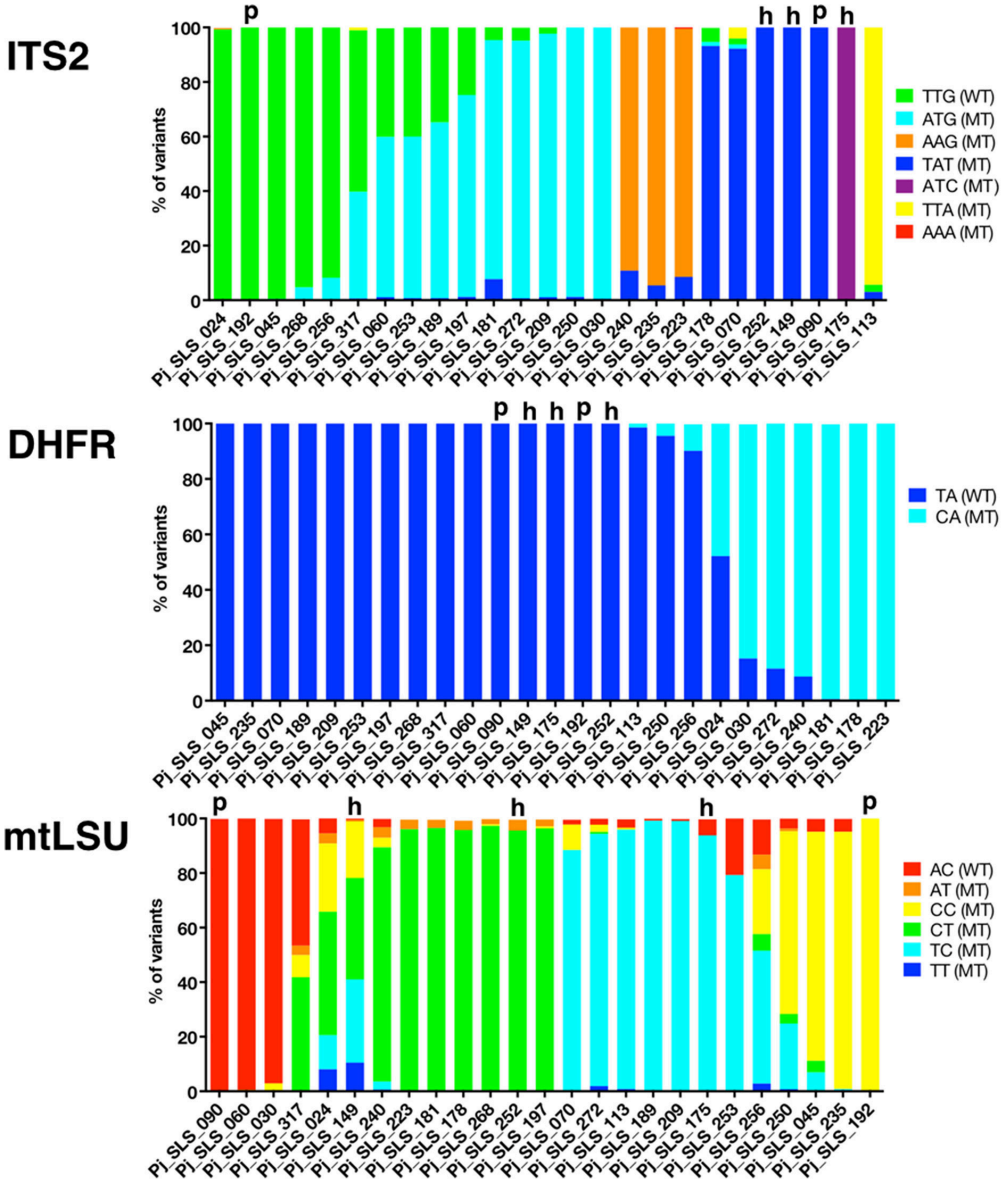
**Proportion of each variant observed in 25 samples, by UDPS, for the mtLSU, ITS2, and DHFR DNA targets**. The proportion of each variant after pyrosequencing analysis (AVA software, Roche) is depicted for each target DNA region, ITS2, DHFR, mtLSU, and for the 25 baseline samples. Each variant is represented by a different color and samples are grouped to allow the visualization of clusters based on the proportion of the variants (this grouping differs for the three targets). Pure samples (“p”) are shown for each target. Pure sequences (proportion of 100%) were observed in 5/25 samples for ITS2 (20%), 17/25 samples for DHFR (68%), and 2/25 samples for mtLSU (8%). The number of times each variant was recovered and the proportions of the variants differed between samples, with some clusters observed. Based on the combined data for the two nuclear DNA targets (ITS2, DHFR), pure sequences were observed in 5/25 (20%) samples (samples 090, 149, 175, 192, 252). Two of these samples were also identified as pure (“p”) for the mtLSU DNA target (samples 090 and 192), whereas the other three samples harbored mixtures of sequences for mtLSU, consistent with mitochondrial heteroplasmy (“h”). Variants accounting for < 0.5% of all sequences and variants with unknown polymorphisms were not considered in this study.

We avoided bias in the interpretation of diversity, by analyzing the proportions of the variants in the 25 baseline samples, as repeat samples were available for six of the 25 patients. Overall, the number of different variants (Table 1, Figure 2) and the proportion of each variant (Figure 3) in each sample differed.

**Table 1:** **Number of variants observed and resulting classifications of each sample for the three DNA target regions analyzed using UDPS and the six STR markers**.

**Sample N°**	**Number of variants**									**Nuclear genome UDPS**	**Nuclear genome UDPS + STR**	**Mitochondrial genome**
	**UDPS**			**STR typing nuclear markers**								
	**Mitochodrial**	**Nuclear**					
	**mtLSU**	**ITS2**	**DHFR**	**022**	**108**	**138**	**189**	**278**	**279**			
Pj_SLS_024	6	2	2	1	1	2	2	1	1	Mixed	Mixed both	Mixed
Pj_SLS_240	6	2	2	1	1	1	2	1	3	Mixed	Mixed both	Mixed
Pj_SLS_250	6	2	2	2	1	1	3	1	1	Mixed	Mixed both	Mixed
Pj_SLS_256	6	2	2	1	1	3	3	2	2	Mixed	Mixed both	Mixed
Pj_SLS_272	5	3	2	2	1	2	3	1	1	Mixed	Mixed both	Mixed
Pj_SLS_045	4	2	1	1	2	2	2	1	1	Mixed	Mixed both	Mixed
Pj_SLS_317	4	4	1	1	1	2	2	1	1	Mixed	Mixed both	Mixed
Pj_SLS_070	3	4	1	1	1	2	2	1	2	Mixed	Mixed both	Mixed
Pj_SLS_235	3	2	1	1	1	1	2	1	1	Mixed	Mixed both	Mixed
Pj_SLS_253	3	3	1	1	1	2	2	1	1	Mixed	Mixed both	Mixed
Pj_SLS_030	2	2	2	1	1	1	2	1	1	Mixed	Mixed both	Mixed
Pj_SLS_181	2	3	2	1	2	1	1	1	1	Mixed	Mixed both	Mixed
Pj_SLS_189	2	3	1	1	1	3	1	1	1	Mixed	Mixed both	Mixed
Pj_SLS_209	2	3	1	1	1	2	1	1	3	Mixed	Mixed both	Mixed
Pj_SLS_060	2	3	1	1	1	1	1	1	1	Mixed	Mixed UDPS	Mixed
Pj_SLS_113	4	3	2	1	1	1	1	1	1	Mixed	Mixed UDPS	Mixed
Pj_SLS_178	2	3	1	1	1	1	1	1	1	Mixed	Mixed UDPS	Mixed
Pj_SLS_197	3	3	1	1	1	1	1	1	1	Mixed	Mixed UDPS	Mixed
Pj_SLS_223	2	3	1	1	1	1	1	1	1	Mixed	Mixed UDPS	Mixed
Pj_SLS_268	3	2	1	1	1	1	1	1	1	Mixed	Mixed UDPS	Mixed
Pj_SLS_149	6	1	1	1	2	1	1	1	1	Pure	Mixed STR	Heteroplasmy
Pj_SLS_175	2	1	1	1	1	1	2	1	1	Pure	Mixed STR	Heteroplasmy
Pj_SLS_252	3	1	1	1	1	1	1	1	1	Pure	Pure	Heteroplasmy
Pj_SLS_090	1	1	1	1	1	1	1	1	1	Pure	Pure	Pure
Pj_SLS_192	1	1	1	1	1	1	1	1	1	Pure	Pure	Pure

### Variation of the number and proportion of variants, revealing possible heteroplasmy

The distribution of the number of variants observed differed significantly between mtLSU, ITS2 and DHFR (Kruskal-Wallis, *p* < 0.0001, Figure 2A, Figure S4A). Up to four, two and six variants/sample were observed for ITS2, DHFR, and mtLSU, respectively. No technical bias was observed, because there was no significant correlation (*p* > 0.05) between the number of variants and fungal load (based on quantification cycle or Cq value) or total number of reads sequenced (Figure S4B). Clusters of samples based on the number of variants for the three targets could be defined (Table 1, Figure 2B). These clusters were not significantly associated with the patient's background (*p* > 0.05).

**Figure 4:**
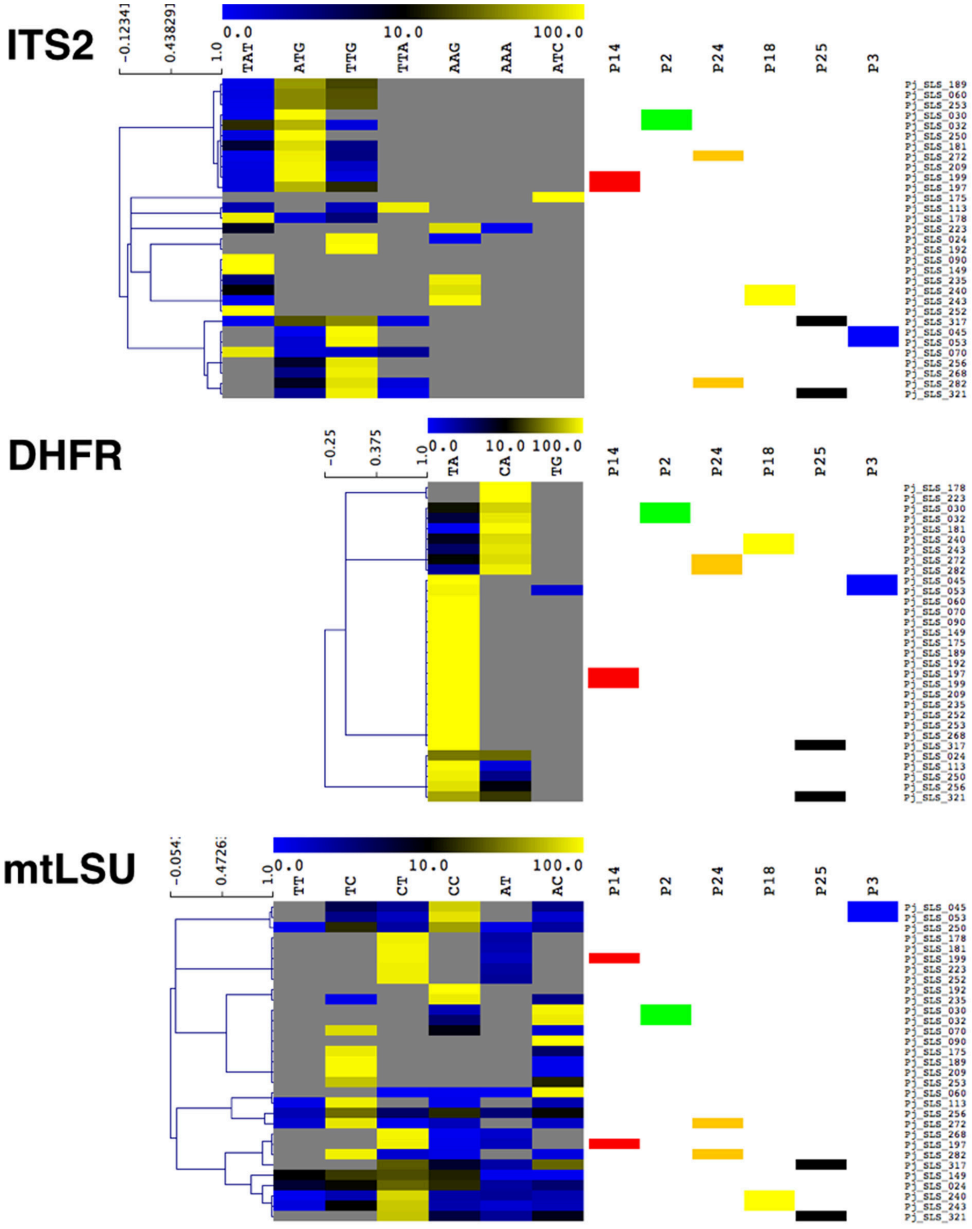
**Heatmap of the proportions of the different variants in the 31 samples analyzed by UDPS, for the mtLSU, ITS2, and DHFR DNA targets**. The 31 samples were clustered by hierarchical clustering analysis based on the proportions of the variants recovered. Samples from the six patients from whom repeat samples were obtained are indicated by a colored box (P14 in red, P2 in green, P24 in orange, P25 in black, and P3 in blue). The clustering analysis showed that, for P2, P18, and P3, the proportion of variants varied little between the two samples, which clustered together. By contrast, for P14, P24, and P25, significant differences in the proportions of variants were observed, leading to the two samples from the same patient being assigned to different clusters. Gray indicates an absence of recovery, blue indicates a low proportion (< 10%) and yellow indicates a high proportion (>70%), as indicated by the scale bar at the top of each heatmap.

The proportion of the total number of reads sequenced for each target corresponding to each of the variants was determined for each of the 25 samples (Figure 3). Samples were classified as pure (single variant on UDPS analysis) or mixed (≥2 variants) sequences. Pure sequences were observed in only 5/25 samples for ITS2 (20%), 17/25 samples for DHFR (68%), and 2/25 (8%) of samples for mtLSU (Table 1, Figure 3). Some of the samples could be clustered on the basis of the proportions of variants, but the pattern of clustering differed between target genes (Figure 4).

Based on the combined UDPS data for the two nuclear target genes (ITS2, DHFR), pure sequences were observed in 5/25 (20%) patients (samples #090, 149, 175, 192, 252) (Figure 2). The genotyping results obtained with the six STR nuclear markers (Gits-Muselli et al., 2015) were consistent with purity (a single allele detected for each of the six markers) for three of these five samples (#090, 192, 252) (Table 1). Two of these five samples (#090, 192) harbored a single mtLSU variant. The other three (#252, 149, 175) had mixed mtLSU sequences, with three, six and two mtLSU variants, respectively (Table 1). These results (polymorphic mtLSU sequences with unique nuclear markers) were suggestive of heteroplasmy.

### Homing endonuclease may be present in *Pneumocystis jirovecii*

**Figure 5:**
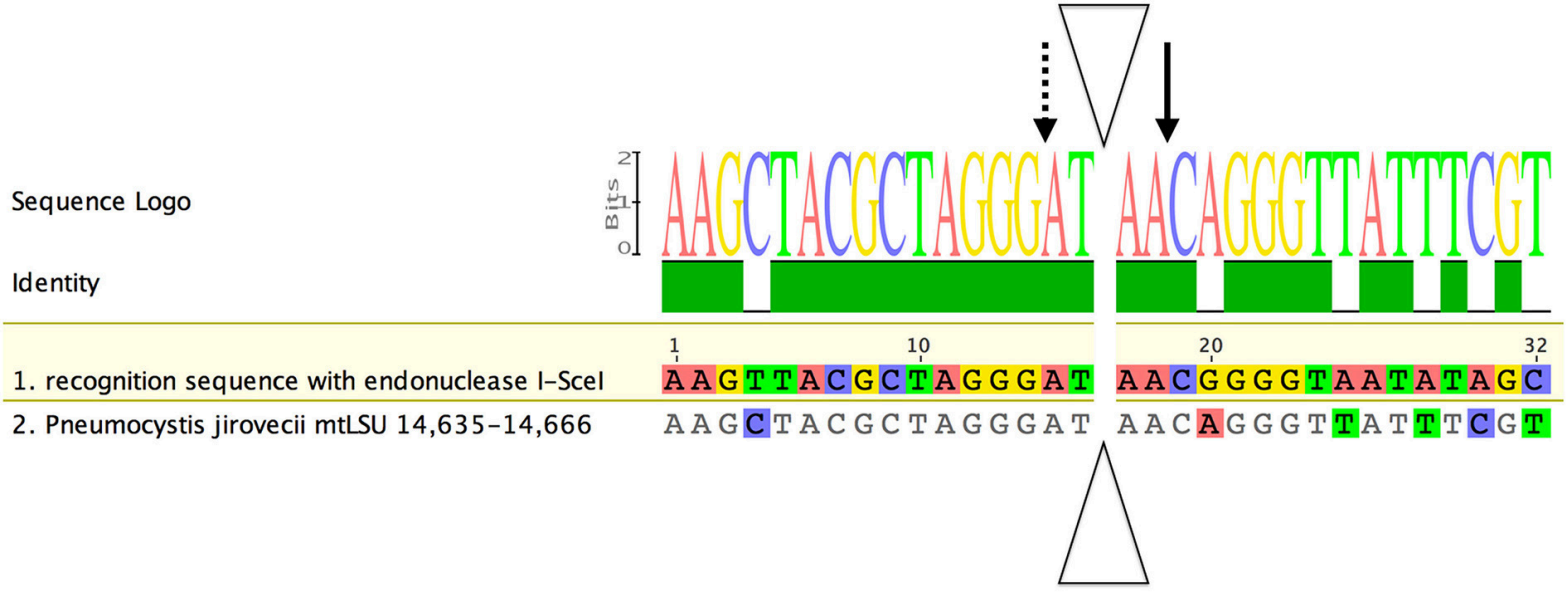
**Alignment of the recognition site for the homing endonuclease I-SceI with the corresponding region in the ***P. jirovecii*** mtLSU gene**. The I-SceI homing endonuclease has a specific recognition site for double-strand DNA break and intron introduction. A putative recognition site for this enzyme was identified between positions 14,635 and 14,666 (JX499143). Nucleotides identical to the consensus sequence are shown in gray, whereas polymorphic positions are shown in color. Solid- and dotted-line arrows indicate cleavages on the presented and complementary strands, respectively. The triangle indicates the intron insertion site (adapted from Belfort and Roberts, 1997).

We searched for the presence of a homing endonuclease site in the mtLSU sequence, because such heteroplasmy could have been acquired through mtDNA recombination events initiated by intron homing (Haugen et al., 2005). Analysis of the complete mtLSU sequence (JX499143) revealed the presence of a recognition site 81.25% identical to that described for the I-SceI of *Saccharomyces cerevisiae* (Dujon, 1980; Jacquier and Dujon, 1985). Only six of the 32 nucleotides differed from those present in the motif described for I-SceI (Figure 5) (Belfort and Roberts, 1997). The two conserved motifs (GIGLILGDA and LAYWFMDDG) of I-SceI were not found in the mtLSU DNA sequence, even if we used the motifs present in *T. deformans* (IVGLMLGDGY and LAVWISDDG), which, like *Pneumocystis*, belongs to the Taphrinomycotina subphylum (Cissé et al., 2014; Almeida et al., 2015).

### Patient-based analysis of *P. jirovecii* diversity

The characteristics of the 25 patients are summarized in Table 2. Sex ratio was 7.3 and median age was 54 years. The patients were HIV-positive (*n* = 15), patients with hematological malignancies (*n* = 7), renal transplant recipients (*n* = 4), and had other causes of immunosuppression (*n* = 5). The country of birth was recovered in 21 patients and was France (*n* = 14), Haiti (*n* = 2), North Africa (*n* = 2), Romania (*n* = 1), Vietnam (*n* = 1), Comoro Islands (*n* = 1). In total, no significant correlation (*p* > 0.05) was found between the presence of the different variants for each gene or for the combination of the three genes and the gender, the age, the background, the country of birth of the patients and the evolution at month 3.

**Table 2:** **Clinical characteristics of the 25 patients sampled in this study**.

**Patient N°**	**Sex**	**Age**	**Country of birth**	**Background**	**CD4 count (/mm^3^)**	**Prophylaxis**	**Treatment**	**Outcome at month 3**
P1	M	32	Na	HIV	48	No	Cotrimoxazole	Alive
P2	M	50	Haiti	HIV	81	No	Cotrimoxazole	Alive
P3	M	10	Na	Systemic lupus erythematosus	na	na	na	na
P4	M	47	Metropolitan France	HIV	216	No	Cotrimoxazole	Alive
P5	F	20	Metropolitan France	Lymphoma	na	Pentacarinat	Atovaquone	Alive
P6	M	65	Vietnam	HIV	2	No	Pentacarinat	Alive
P7	M	45	North Africa	Kidney SOT	170	No	Cotrimoxazole	Alive
P8	M	45	Metropolitan France	HIV	2	No	Cotrimoxazole	Alive
P9	M	42	Metropolitan France	Lymphoma	na	No	Cotrimoxazole	Alive
P10	M	48	Na	HIV	na	na	na	na
P11	M	56	Metropolitan France	Kidney SOT	na	No	Cotrimoxazole	Alive
P12	F	64	Metropolitan France	Kidney SOT	59	No	Cotrimoxazole	Alive
P13	M	33	Metropolitan France	HIV	12	No	Cotrimoxazole	Alive
P14	M	69	Comores	Lymphoma	11	No	Cotrimoxazole	Death
P15	M	66	North Africa	HIV	116	No	Cotrimoxazole	Alive
P16	M	54	Haiti	Kidney SOT	268	No	Cotrimoxazole	na
P17	M	59	Metropolitan France	HIV	20	No	Cotrimoxazole	Alive
P18	M	57	Na	Lung cancer	na	No	Cotrimoxazole	Alive
P19	M	0.3	Metropolitan France	Neonate	na	na	na	na
P20	M	50	Metropolitan France	HIV	38	No	Cotrimoxazole	Alive
P21	M	81	Metropolitan France	Lymphoma	na	No	Cotrimoxazole	Alive
P22	M	56	Metropolitan France	Lymphoma	256	No	Cotrimoxazole	Alive
P23	F	57	Metropolitan France	HSCT and Lymphoma	9	Atovacone	Cotrimoxazole	Death
P24	M	62	Metropolitan France	HIV	56	No	Cotrimoxazole	Alive
P25	M	60	Roumania	HIV	10	No	Cotrimoxazole	Alive

*na: not available*.

For the six patients with repeated samples (median time between the first and second samples: 7.5 days, range: 1–18 days), the proportions of the variants varied by more than 2% (cutoff for reproducibility) in all patients, and by more than 10% in two patients (P14, P25) (Table 3). In addition to the differences in the proportions of the variants present, some of the variants were present in one sample, and absent from the other in five patients (P2, P3, P14, P24, and P25), although the proportion of this variant was below 2% when it was present in some cases (P2, P3, P14, P24). Even for samples recovered only a few days apart (2 days for P2, and 1 day for P14), the proportions of the variants varied by up to 24.9% between samples (ITS2 and TAT for P2, ITS2 and TTG for P14) (Table 3). In P25, one DHFR variant (CA) accounted for 30.4% of the variants present 8 days after the first sample was collected and cotrimoxazole treatment was initiated (Table 3). Clustering on the basis of the proportion of variants for the three targets revealed that the two samples from P2, P3, and P18 clustered together, whereas those from P14, P24, and P25 did not (Figure 4).

**Table 3:** **Description of the proportion of variants in the repeated samples from six patients based on three DNA target regions analyzed using UDPS**.

**Patient N°**	**Sample N°**	**Specimen**	**Delay from first sample (days)**	**mtLSU variants (%)**						**ITS2 variants (%)**							**DHFR variants (%)**		
				**TT**	**TC**	**CT**	**CC**	**AT**	**AC**	**TAT**	**ATG**	**TTG**	**TTA**	**AAG**	**AAA**	**ATC**	**TA**	**CA**	**TG**
P2	Pj_SLS_030	Sputum					2.9		97.0	0.5	99.5						15.2	84.5	
	Pj_SLS_032	BAL	+2				5.6		94.3	25.4	73.4	1.3					7.5	92.5	
P3	Pj_SLS_045	BAL			6.9	4.3	84.0		4.7		0.5	99.5					100.0		
	Pj_SLS_053	BAL	+14		4.6	2.5	91.1		1.8		1.7	97.9					96.5		1.7
P14	Pj_SLS_197	Sputum				96.4	0.7	2.5		1.2	74.1	24.8					100.0		
	Pj_SLS_199	BAL	+1			96.8		2.5		1.4	97.5	1.2					100.0		
P18	Pj_SLS_240	Sputum		0.6	3.0	85.9	3.5	3.9	3.0	10.8				89.2			8.7	88.0	
	Pj_SLS_243	Sputum	+7	1.2	10.0	81.2	3.1	1.8	2.7	0.8				99.3			6.1	91.3	
P24	Pj_SLS_272	Sputum		1.9	92.5	0.7	2.6		2.2	0.7	94.4	4.7					11.6	88.4	
	Pj_SLS_282	BAL	+18		95.9	1.7	1.0		1.1	8.8	89.8	1.4					4.1	94.7	
P25	Pj_SLS_317	Sputum				41.8	8.1	3.5	46.3	0.6	39.1	59.2	1.0				100.0		
	Pj_SLS_321	BAL	+8			79.5	7.4	4.0	8.9		4.3	94.9	0.7				68.3	30.4	

*BAL, bronchoalveolar lavage fluid; mtLSU GenBank ID, JX499143; ITS2 GenBank ID, JQ365725; DHFR GenBank ID, AF090368*.

## Discussion

Our next-generation sequencing results indicate that *Pneumocystis* infections are mostly mixed infections. UDPS of nuclear gene targets revealed that 80% of the samples harbored mixed sequences, and this proportion increased to 92% (23/25) when a mitochondrial DNA target was added to the analysis.

Primary infection occurs early in life, with about 85% of children displaying anti-*Pneumocystis* antibody-mediated immune responses by the age of 18 months (Meuwissen et al., 1977; Pifer et al., 1978) and the detection of fungal DNA in non-invasive respiratory samples (Vargas et al., 2001). Immunocompetent or immunosuppressed individuals act as a reservoir for *P. jirovecii*, which infects immunocompromised patients via airborne transmission (Gigliotti et al., 2003; Chabé et al., 2004; Choukri et al., 2010), as demonstrated by epidemiological studies of numerous PCP outbreaks in a context of intrahospital transmission, particularly in renal transplant units (de Boer et al., 2011). The high predominance of mixed infection observed in our study may reflect continuous exposure throughout life (Gigliotti and Wright, 2012), together with the active multiplication of a subset of strains during immunosuppression. Some strains may be less capable of active multiplication than others in specific settings and in a given patient. This does not exclude the possibility that reactivation from dormancy contributes to the diversity observed during infection, because differences have been observed in the geographic distributions of *P. jirovecii* genotypes (Alanio et al., 2015). From an epidemiologic standpoint, these findings demonstrate that mitochondrial genes, including the mtLSU gene in particular, should be used with caution for the MLST genotyping of *P. jirovecii* because the mitochondrial and nuclear genomes may evolve differently.

Different mechanisms are thought to be involved in mutations of the mitochondrial genome. Mitochondrial DNA is subjected to more rounds of replication than nuclear DNA, and this may result in a higher frequency of point mutations. Mutations may also accumulate more easily due to higher levels of exposure to reactive oxygen species and, finally, the DNA repair machinery of the mitochondria has been shown to be less efficient than that of the nucleus (Song et al., 2005). Interestingly, five samples harbored pure nuclear (ITS and DHFR) sequences but mixed mitochondrial sequences, suggesting that heteroplasmy had occurred. Of course, we cannot completely rule out the possibility of polymorphism in other nuclear sequences. However, the analysis of six additional nuclear markers by STR typing (high level of discrimination and widespread distribution within the *P. jirovecii* genome; Gits-Muselli et al., 2015) was consistent with purity (unique genotype) for three samples. Heteroplasmy has already been reported in fungi, mostly for filamentous fungi (Barr et al., 2005). However, heteroplasmy seems to be rare in most fungi, due to uniparental mitochondrial transmission (Barr et al., 2005). In laboratory conditions, heteroplasmy is controlled and used to study mtDNA recombination (Basse, 2010). By contrast, heteroplasmy has rarely been studied in natural populations. Mitochondrial gene variation between isolates has been demonstrated by cloning in arbuscular mycorrhizal fungi (*Rhizophagus irregularis*) (Beaudet et al., 2015), with the identification of up to 20 variants per isolate for various mitochondrial target regions, all different from mtLSU. This aspect has been studied in natural populations of only two medically important fungi, *Candida albicans* (Anderson et al., 2001) and *Cryptococcus gattii* (Xu et al., 2009), to our knowledge. In both cases, the authors used PCR followed by Sanger sequencing and strain culture, assuming that the culture was clonal and therefore had only one nuclear genome. As expected, given the greater sensitivity of UDPS, we detected a higher degree of polymorphism within *P. jirovecii* mtLSU. Moreover, we had to control for the uniqueness of the nuclear genome in the absence of a simple culture system. Heteroplasmy was, therefore, expected in UDPS with a 0.5% cutoff value for variant selection. The uncultivable nature of *P. jirovecii* complicates studies of this phenomenon and the interpretation of its importance. However, recently developed culture methods may facilitate studies of the dynamics of heteroplasmy (Schildgen et al., 2014). However, homoplasmy is widely described in various organisms and controlled by genetic mechanisms (Christie et al., 2015). The observation of heteroplasmy in *P. jirovecii* could be the consequence of the lack of the control mechanisms that tend to decrease heteroplasmy rates are not present in *P. jirovecii*, or their ineffectiveness during the active phase of disease. *P. jirovecii* could also regularly or occasionally inherit mitochondria from both parents. In addition, a genetic phenomenon called intron homing responsible for recombination in the mitochondrial genome could lead to heteroplasmy.

Homing endonuclease genes (HEGs) are widespread in fungal genomes and are generally located in self-splicing introns (Basse, 2010). A recognition site for the homing endonuclease I-SceI was found in the *S. cerevisiae* mtLSU gene (Dujon, 1980; Jacquier and Dujon, 1985). This suggests that heteroplasmy in *P. jirovecii* may result from recombination due to the double-strand breaks created by such enzymes in mitochondrial DNA. However, the classical LAGLIDADG (Belfort and Roberts, 1997) motif and the conserved motifs GIGLILGDA and LAYWFMDDG (Chevalier et al., 2005) were not detected in the mtLSU gene, even if the mitochondrial homolog from *T. deformans* (the closest relative of *Pneumocystis*; Cissé et al., 2013, 2014; Almeida et al., 2015) was used as the reference (GenBank: CAUL01000001). This suggests that there may be as yet undiscovered homologs of I-SceI endonucleases in *P. jirovecii*.

Several variants, including those of the mtLSU target, were clearly present at the time of disease diagnosis, when fungal load was high. The observed diversity may also be due to mutations acquired during active growth of the fungus during immunosuppression and in the days or weeks before diagnosis. We cannot exclude the possibility of a peculiar variant having a selective advantage over another variant, resulting in changes in their relative proportions overtime. A stable final equilibrium in the proportion of variants may be reached in some patients, but not in others. Indeed, we observed variations in the proportions of variants in analyses of results for the serial samples obtained from some patients. This variation may also be due to differences in the sampling method, because *P. jirovecii* isolates recovered from BAL or sputum have already been shown to be different from those recovered from lung tissue (Helweg-Larsen et al., 2001). One patient (P25) with AIDS acquired a mutation of the DHFR target DNA (T192C) in about 30% of all DHFR sequences 8 days after the initiation of cotrimoxazole treatment (Table S1). This mutation corresponds to a T1158C synonymous substitution (called T312C in Nahimana et al., 2004) with no effect on protein function and not reflecting the acquisition of a resistance mutation. The other coding regions of this gene have not been studied and may contain other mutations. However, the observed variant accounted for only 30% of all variants, and complete recovery with no clinical resistance to cotrimoxazole was observed. The detection of this variant only 8 days after the introduction of cotrimoxazole treatment could suggest acquisition of a mutation during proliferation in the presence of the drug or selection and proliferation of this under treatment pressure.

Our study provides additional evidence for the complex natural history of *P. jirovecii* infections, for which the three Henle-Koch's postulates long used to evaluate the causal relationship between an infectious agent and a clinically defined disease can't be applied (Evans, 1976; Falkow, 1988). We show that these infections are mostly associated with mixtures of strains/genotypes that were probably acquired over the course of the patient's life. This finding is consistent with the current view of the natural history of these infections: reservoir in individual with various backgrounds (immunocompetent infants and adults or individuals with chronic pulmonary diseases such as chronic obstructive pulmonary disease or with relative immunosuppression such as children and pregnant women; Peterson and Cushion, 2005) and direct airborne transmission (Cushion, 2010; Gigliotti and Wright, 2012). However, we still know little about the subjects carrying *P. jirovecii* but not developing active infection (Hauser et al., 2000; Morris and Norris, 2012), as the low fungal load in respiratory samples from these individuals precludes such analyses, particularly for single-copy nuclear DNA targets.

## Author contributions

Conceived and designed the experiments: AA, SB. Performed the experiments: MG, SM. Analyzed the data: AA, FD, SB. Contributed reagents/materials/analysis tools: SM, FD. Wrote the paper: AA, FD, SB. Major criticism of the manuscript: FD, SB.

### Conflict of interest statement

The authors declare that the research was conducted in the absence of any commercial or financial relationships that could be construed as a potential conflict of interest.

## Supplementary material

Figure S1 **Alignment of ITS2 sequences recovered from GenBank (***n*** = 120) (A) and of the amplicons recovered from our patients, with the polymorphic bases highlighted (B)**.

Figure S2 **Alignment of DHFR sequences recovered from GenBank (***n*** = 60) (A) and of the amplicons recovered from our patients, with the polymorphic bases highlighted (B)**.

Figure S3 **Alignment of mtLSU sequences recovered from GenBank (***n*** = 52) (A) and of the amplicons recovered from our patients, with the polymorphic bases highlighted (B)**.

Figure S4 **Distribution of the number of variants found in each sample for mtLSU, ITS2, and DHFR DNA targets, as determined by UDPS and with six nuclear STR markers (A)**. Distribution of fungal load (left panel) and the number of reads for each patient (one dot per patient, *n* = 31), classified by the maximum number of variants recovered per sample (1–4 for ITS, 1–2 for DHFR, and 1–6 for mtLSU) **(B)**.

Table S1 **Correspondence between the position of the observed polymorphic bases described in our samples and their position in the reference sequence, with the corresponding amino acid substitution**.

Table S2 **Primers and PCR conditions used**.
