# Supplementary figures and images for: Diversity of Pneumocystis jirovecii during Infection Revealed by Ultra-Deep Pyrosequencing
Source: Front Microbiol. 2016 May 24;7:733. doi: 10.3389/fmicb.2016.00733 (PMC4877386; doi:10.3389/fmicb.2016.00733)

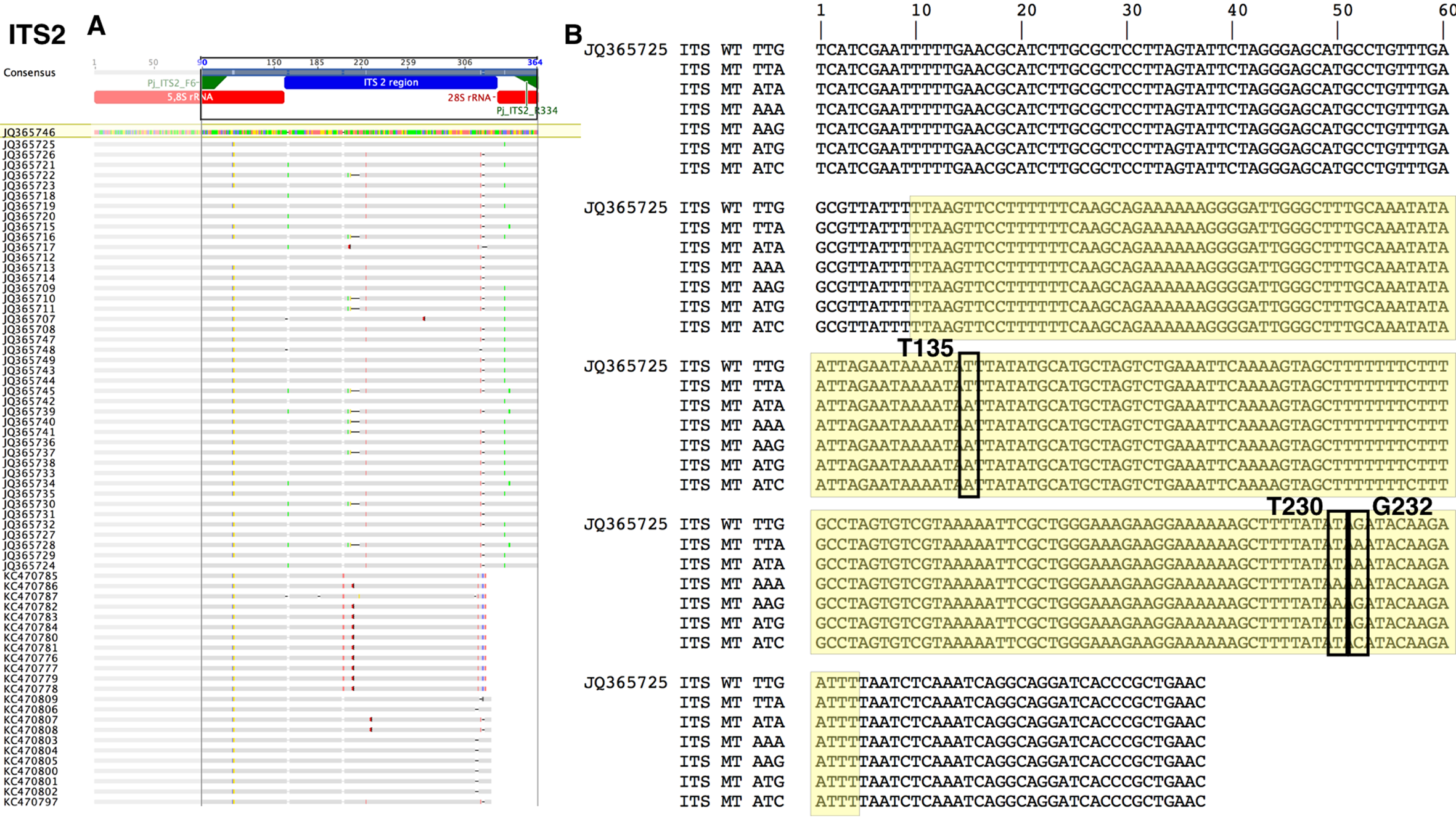

Supplement: Figure S1 — Alignment of ITS2 sequences recovered from GenBank (n = 120) (A) and of the amplicons recovered from our patients, with the polymorphic bases highlighted (B). [file Image1.tiff]

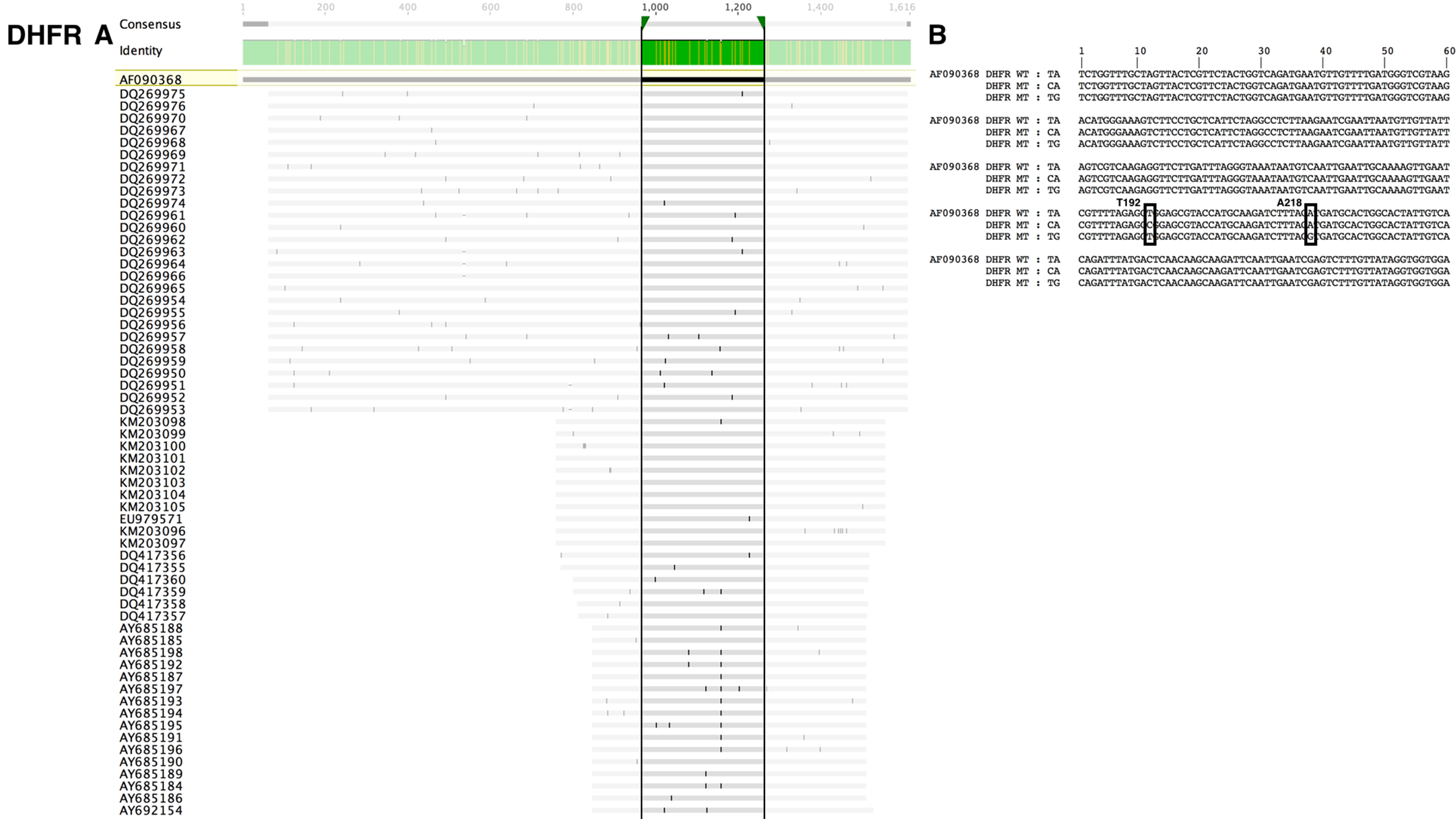

Supplement: Figure S2 — Alignment of DHFR sequences recovered from GenBank (n = 60) (A) and of the amplicons recovered from our patients, with the polymorphic bases highlighted (B). [file Image2.TIFF]

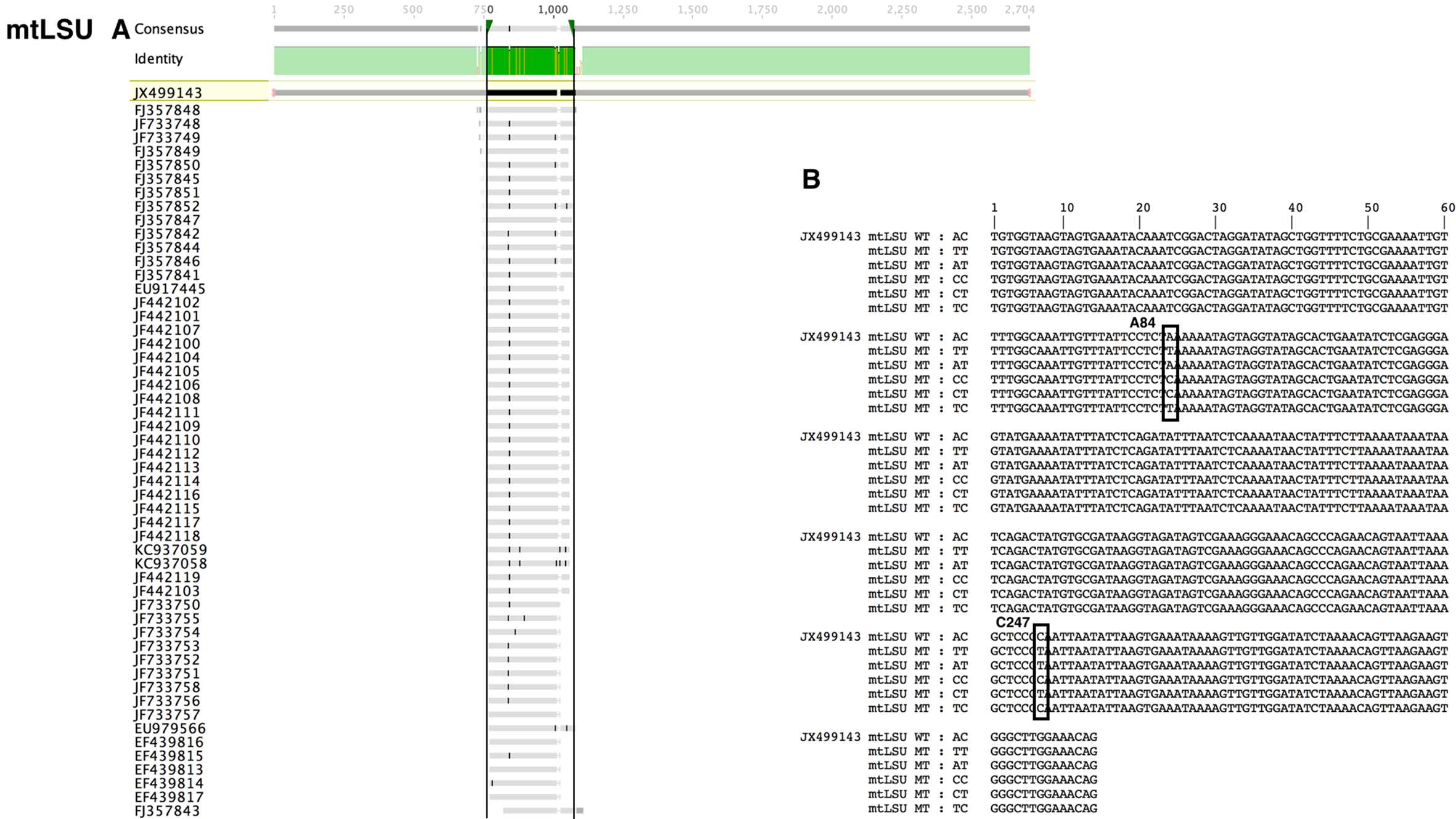

Supplement: Figure S3 — Alignment of mtLSU sequences recovered from GenBank (n = 52) (A) and of the amplicons recovered from our patients, with the polymorphic bases highlighted (B). [file Image3.TIFF]

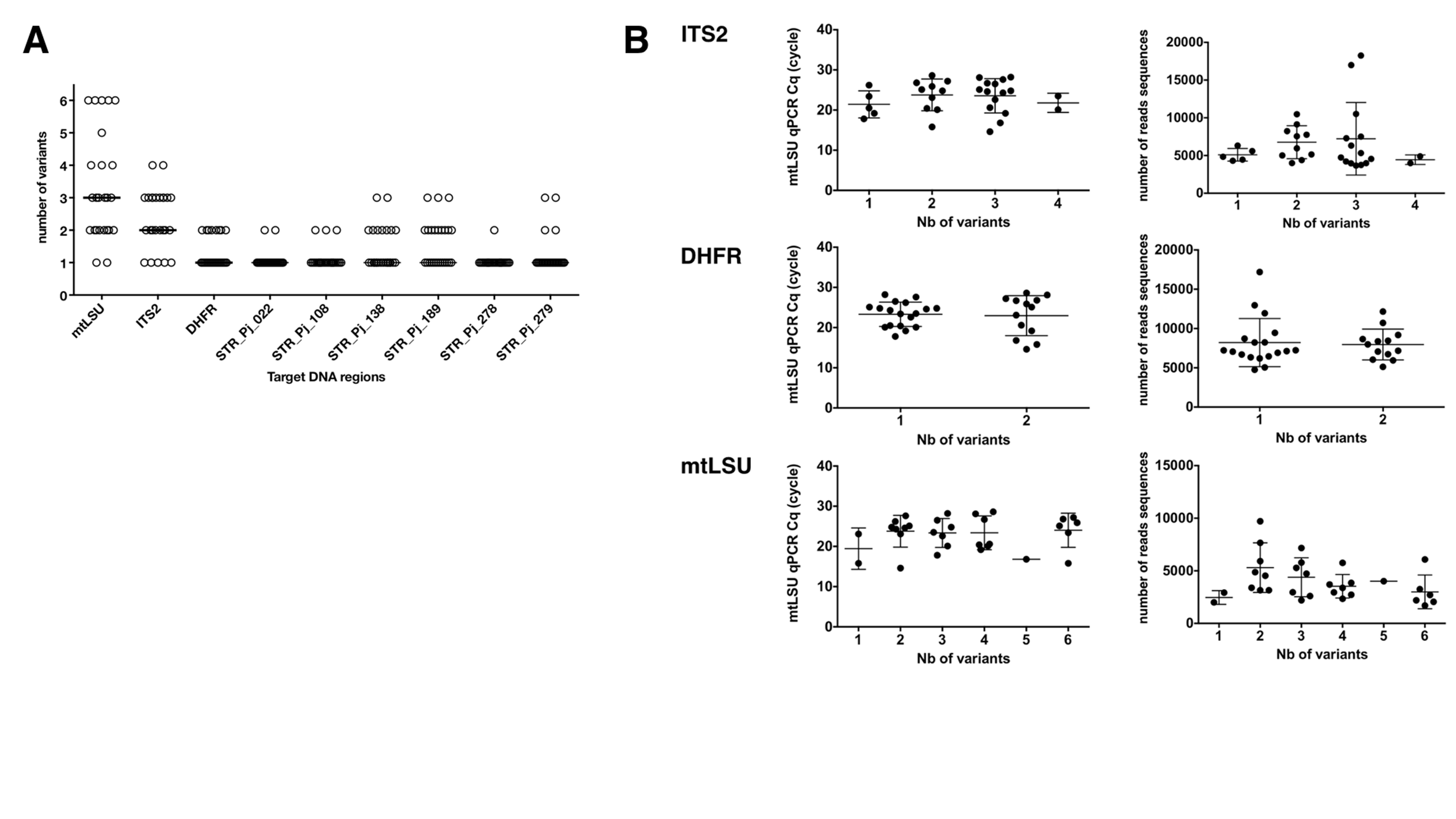

Supplement: Figure S4 — Distribution of the number of variants found in each sample for mtLSU, ITS2, and DHFR DNA targets, as determined by UDPS and with six nuclear STR markers (A). Distribution of fungal load (left panel) and the number of reads for each patient (one dot per patient, n = 31), classified by the maximum number of variants recovered per sample (1–4 for ITS, 1–2 for DHFR, and 1–6 for mtLSU) (B). [file Image4.TIFF]
