# Supplementary material for: Diversity of Pneumocystis jirovecii during Infection Revealed by Ultra-Deep Pyrosequencing
Source: Front Microbiol. 2016 May 24;7:733. doi: 10.3389/fmicb.2016.00733 (PMC4877386; doi:10.3389/fmicb.2016.00733)
Supplement: Table S1 — Correspondence between the position of the observed polymorphic bases described in our samples and their position in the reference sequence, with the corresponding amino acid substitution. [file Table1.docx]

Table S1 : Correspondence between the position of the observed polymorphic bases described in our samples and the position of the same polymorphic bases in the reference sequence with the corresponding amino acid substitution

|  | Correspondence to reference sequence | Corresponding aminoacid substitution |
| --- | --- | --- |
| ITS2_277bp amplicon | JQ365725 |  |
| T135A | T408A | / |
| T230A | T503A | / |
| G232A | G505A | / |
| G232C | G505G | / |
| DHFR_300pb amplicon | AF090368 |  |
| T192C | T1158C | synonymous |
| A218G | A1183G | D99G |
| mtLSU_314bp amplicon | JX499143 |  |
| A84T | A13,215T | / |
| A84C | A13,215C | / |
| C247T | C13,378T | / |
