# Supplementary material for: Diversity of Pneumocystis jirovecii during Infection Revealed by Ultra-Deep Pyrosequencing
Source: Front Microbiol. 2016 May 24;7:733. doi: 10.3389/fmicb.2016.00733 (PMC4877386; doi:10.3389/fmicb.2016.00733)
Supplement: Table S2 — Primers and PCR conditions used. [file Table2.docx]

| Primer Name | Primer sequence | Tm (°C) | Amplicon lenght  (bp) | Primer final concentration (µM) | MgCl2 concentration (mM) |
| --- | --- | --- | --- | --- | --- |
| mtLSU_35F | TGTGGTAAGTAGTGAAATACAAATCGG | 54 | 314 | 0.2 | 3 |
| mtLSU_329R | CTGTTTCCAAGCCCACTTCT |  |  |  |  |
| ITS_6F | TCATCGAATTTTTGAACGCAT | 54 | 277 | 0.2 | 3 |
| ITS_334R | GTTCAGCGGGTGATCCTG |  |  |  |  |
| DHFR_967F | TCTGGTTTGCTAGTTACTCG | 55 | 300 | 0.2 | 3 |
| DHFR_1266R | TCCACCACCTATAACAAAGAC |  |  |  |  |

Table S2: Primers and PCR conditions used in this study
